# Bioactive Peptides: Synthesis, Sources, Applications, and Proposed Mechanisms of Action

**DOI:** 10.3390/ijms23031445

**Published:** 2022-01-27

**Authors:** Mohsen Akbarian, Ali Khani, Sara Eghbalpour, Vladimir N. Uversky

**Affiliations:** 1 Department of Chemistry, National Cheng Kung University, Tainan 701, Taiwan; mohsen.akbarian66@gmail.com; 2 Department of Radiation Sciences, Faculty of Applied Medicine, Iran University of Medical Sciences, Tehran 1449614535, Iran; khaniali1980rad@gmail.com; 3 Department of Obstetrics and Gynecology Surgery, Babol University of Medical Sciences, Babol 4717647745, Iran; saraeghbalpour23@yahoo.com; 4 Department of Molecular Medicine and Health Byrd Alzheimer’s Institute, Morsani College of Medicine, University of South Florida, Tampa, FL 33612, USA

**Keywords:** bioactive peptides, production of peptides, application of peptides, mechanism of application

## Abstract

Bioactive peptides are a group of biological molecules that are normally buried in the structure of parent proteins and become active after the cleavage of the proteins. Another group of peptides is actively produced and found in many microorganisms and the body of organisms. Today, many groups of bioactive peptides have been marketed chemically or recombinantly. This article reviews the various production methods and sources of these important/ubiquitous and useful biomolecules. Their applications, such as antimicrobial, antihypertensive, antioxidant activities, blood-lipid-lowering effect, opioid role, antiobesity, ability to bind minerals, antidiabetic, and antiaging effects, will be explored. The types of pathways proposed for bioactive applications will be in the next part of the article, and at the end, the future perspectives of bioactive peptides will be reviewed. Reading this article is recommended for researchers interested in various fields of physiology, microbiology, biochemistry, and nanotechnology and food industry professionals.

## 1. Introduction

The philosophy of science is to improve the quality of human life, and for many years, a large number of people have focused on increasing this quality. Of the previous attempts, using bioactive peptides (BPs) is a particularly promising strategy. These materials, along with their biosafety, have medicinal, cosmetic, and even nutritional properties [1]. BPs are generally a group of peptides, in most cases consisting of fewer than 50 residues, that have a function in a living organism or cell. Although some of these peptides are found in a bare format, many of them are hidden in the intact structure of protein molecules [2]. The contents of BPs’ chains in most cases comprise the amino acid proline, arginine, and lysine, along with hydrophobic residues.

From a structural point of view, there is no consensus on the architecture of BPs [3]. They are classified into two main types: endogenous and exogenous peptides. Endogenous peptides are produced in different types of cells, such as neural cells (analgesic/opioid application) or immune cells (role in inflammation and antimicrobial), or in various glands throughout the body, such as the pituitary and adrenal glands. Exogenous peptides enter the body from various sources, such as foods, dietary supplements, and medications [4]. BPs are specific components that have significant biological effects and have a positive effect on body function or condition. As a result, they have received a lot of attention due to their application in increasing the quality of health and, economically, because of their use in the production of healthy foods, drugs, and other products [5].

The different physiological roles of peptides have made them a good choice for the production of therapeutic compounds. Different types of physiological activity of bioactive peptides have been reported, depending on their type, number, sequence, and the properties of the amino acids [2]. From a nutritional point of view, the bioavailability of peptides is greater than that of proteins. In addition, smaller peptides have less allergenic effects than primary proteins, and as a result, the products of protein hydrolysis are widely used in infant formulas [6].

Therapeutically, there are many benefits from peptides that make them more useful than traditional medicines. For example, bioactive peptides have more specialized activities on the target tissue and therefore have little or no toxic effects; they are also effective even at low concentrations. This feature is operative in treating chronic diseases. On the other side, synthetic chemical compounds that are commonly used as drugs have a cumulative effect on the body. While they are still active, these chemicals may cause environmental problems due to their excretion.

**Figure 1:**
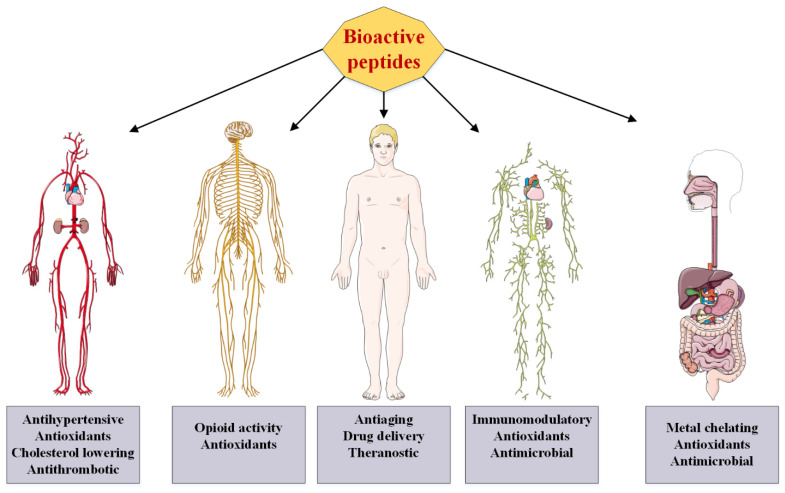
Different applications of bioactive peptides for humans.

Conversely, bioactive peptides have no accumulation in organisms and are easily excreted and destroyed. At first glance, this feature may be considered a negative point, but due to the adverse consequences that may occur in the body from the toxicity of the intake of drugs, timely disposal or destruction of these drugs after use in the body can be considered a very desirable feature [7]. We first study the various methods of producing bioactive peptides and then review the types of sources that these peptides can be obtained from. Next, we examine the types of their applications (summarized in Figure 1) and their mechanisms of action. We conclude with a vision for the future and some strategic suggestions for those interested in these important biological molecules.

## 2. Production of Bioactive Peptides

**Figure 2:**
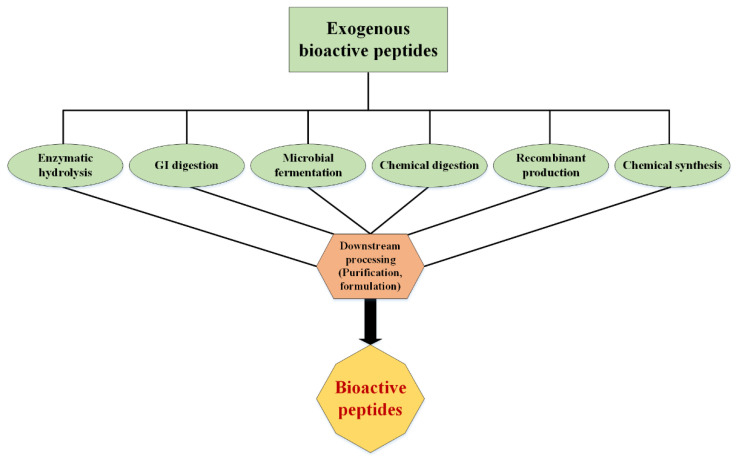
Different pathways for the production of exogenous bioactive peptides.

Various methods have been developed to obtain BPs (Figure 2). The characteristics of an optimal method include low cost, industrialization capability, reproducibility, and biocompatibility. To produce some peptides that have unconventional amino acids or contain specific functional groups, such as fatty acids or sugars, postpurification engineering and/or chemical production are required. These peptides are usually medicinal and less commonly used as food supplements [8].

### 2.1. Enzymatic Hydrolysis

Since most bioactive peptides are buried or encrypted in the structure of mature proteins, the most common and simple method for producing bioactive peptides is enzymatic hydrolysis, especially by digestive enzymes [9]. The use of gastrointestinal enzymes to produce bioactive peptides makes it possible to administer the resulting peptides orally [10]. Once the amino acid sequence of the molecules is known, the peptides may be synthesized by chemical synthesis or recombinant deoxyribonucleic acid (DNA) technology, which will be discussed later. Enzymatic hydrolysis of bioactive peptides can be performed from precursor proteins in three ways: (I) enzymatic hydrolysis by enzymes extracted from microorganisms or plants, (II) enzymatic hydrolysis by digestive enzymes, and (III) microbial fermentation. In some cases, a combination of (I) and (II) or (I) and (III) has been found to be effective in the production of short-chain peptides [11].

The use of specific or even nonspecific proteases is the most common way to produce bioactive peptides, as it takes less time to reach the degree of hydrolysis, and it is possible to better control the hydrolysis process to achieve peptides with specific molecular weights and amino acid composition. In this process, various enzymes, such as pepsin, bromelain, trypsin, chymotrypsin, and papain, are used under their optimum pH and temperature conditions. Many known bioactive peptides are mainly produced using digestive enzymes, including pepsin and trypsin [12]. For example, angiotensin-converting enzyme (ACE) inhibitory peptides and calcium-binding phosphopeptides are commonly produced by trypsin [13]. Additionally, many thermozymes, such as enzymes from bacterial and fungal sources, have been used to produce bioactive peptides. Additionally, several plant-based enzymes, such as papain and pronase, have been applied for enzymatic hydrolysis of soy flour and wheat flour [14].

Many bioactive peptides, such as biogenic, opioid, immunomodulatory, salt/metal-binding, antihypertensive, and antimicrobial peptides, can be produced by enzymatic hydrolysis of foods such as milk, animal and fish meat, corn, wheat, soybeans, and eggs [15]. In industrial-scale conditions, the use of surface-coated enzymes is more common than conventional soluble enzymes. Coated enzymes allow enzymatic hydrolysis under more moderate and controlled conditions. In addition, these fixed enzymes can be recovered to prevent the production of secondary metabolites due to enzyme autolysis [16]. Another common method on an industrial scale is the use of continuous hydrolysis, which is widely used for the complete conversion of food proteins from various sources into hydrolysis products with improved nutritional or functional properties. This method is more efficient and less expensive under industrial conditions than conventional methods and is obtained by using reactors equipped with ultrafiltration membranes with different components, which may be combined with other purification techniques or units [17].

The final product of enzymatic hydrolysis depends on the type of used enzyme, the type of protein precursor, the degree of hydrolysis, and the separation method of the final sample. Although both crude and purified peptides are used for different applications, to reduce the final price, the use of crude types of peptides is more preferred [2]. In some cases, in addition to the physical properties of the peptide, its three-dimensional structure is also important. Some peptides, especially antimicrobial peptides, have a cyclic structure (via disulfide bonds) or beta-sheets on their structure, which are necessary to their functions [18]. During the production of such peptides, the structure of the parent protein must not undergo harsh spatial change. On the other hand, this will be a challenge in the performance of hydrolase enzymes because it may reduce the enzyme’s access to the cleavage site at a particular site of the protein.

Recently, some novel methods have been developed to overcome these conundrums. For example, it has been shown that by applying pressure (above 100 MPa) during enzymatic hydrolysis (high hydrostatic pressure processing, HHP), the efficiency of the enzymes will increase. Furthermore, high pressure has been shown to cause temporary and reversible changes in the protein structure that increase the access of hydrolase to a variety of cutting sites on the protein surface [19,20]. Another study reviews such innovative methods, which generally increase the efficiency of enzymatic hydrolysis during food processing [21].

#### Microbial Fermentation

There is another method, called microbial fermentation, to produce bioactive peptides, which is a type of bacterial hydrolase used to break down proteins into small peptides. In fact, this preparation of peptides is part of the enzymatic hydrolysis method that uses bacteria. Many industrially used primer cultures have high proteolytic potency. Thus, bioactive peptides can be produced by primer and nonprimer bacteria used in fermented food products [21]. *Lactobacillales*, which are a large group of beneficial bacteria in nature and are also found in our digestive system, are used to produce bioactive peptides. Their role in the production of fermented products is not only due to their physiological effect but also due to their technological importance in the development of texture and taste.

The proteolytic systems of *Lactobacillales*, such as *Lactococcus lactis* [22], *Lactobacillus helveticus* [23], and *Lactobacillus delbrueckii* of the *bulgaricus* subspecies [24] are now well known. These systems consist of several proteins attached to the cell wall and several intracellular proteins, including endopeptidases, aminopeptidases, tryptidases, and dipeptidases [25]. Some studies have shown that the use of multiple fermentations, as well as the combination of enzymatic hydrolysis, increases the production of bioactive peptides. A study reported that fermented milk products with a commercial mixed culture primer containing five strains of *Lactobacillales* increase ACE inhibitory activity [26]. Treatment of milk with trypsin before fermentation with yogurt primer cultures results in the formation of phosphopeptide-rich peptides. In these samples, the productions of the calcium phosphopeptides (CPP) β-caseins (β-CN, 1-25-4P) and αs1-casein (αs1-CN) (43-79)-7 were reported, while the amount of proteolysis in samples that were fermented alone was not significant [27].

High inhibitory activity of 2,2′-azino-bis(3-ethylbenzothiazoline-6-sulfonic acid) (ABTS) and 2,2-diphenyl-1-picrylhydrazyl (DPPH) radicals of fermented camel milk with *Leuconostoc lactis* has been reported [28]. In addition to living microorganisms, proteolytic enzymes isolated from *Lactobacillales* have been successfully used to generate bioactive peptides from milk proteins. In one study, the ACE inhibitory activity of the casein hydrolysis product was measured using five different commercial proteolytic enzymes. Among these enzymes, peptides produced from a protease isolated from *Aspergillus aureus* showed the highest inhibitory activity of ACE in vitro compared with other peptides [29]. In comparison with other methods, microbial fermentation is a cheaper way to produce peptides. This is because microorganisms are a cheap source of proteases and are known to be safe. The cost of bacterial cultures is relatively low due to the minimum nutrient requirements and short growth time. Additionally, the proteases of *Lactobacillales* are expressed on the cell wall, which makes extraction protocols relatively easy and inexpensive [30].

### 2.2. Chemical Synthesis of Peptides

In the process of chemical production, the bioactive peptides are produced from amino acid units in a defined chemical environment. To chemical synthesis of bioactive peptides, there are currently two major strategies, namely, soluble-phase and solid-phase syntheses. In addition to these two methods, there is another chemical method, hybrid synthesis, which is sometimes used to produce pharmaceutical peptides. The proportion of the type of the used protecting groups in the structure of amino acids and the method of deprotection is the key step in the synthesis of bioactive peptides [31]. Chemical synthesis of peptides by soluble-phase methods was first used in 1953 to produce pharmaceutical insulin peptides [32]. The basis of this method is the reaction of amino acids in a soluble medium.

Perhaps the most important advantage of soluble-phase methods is the economic justification and purification at each stage of the synthesis since it uses less expensive materials and equipment [33]. In this method, during the production of desired peptides, the side synthesis of intermediates is an undesirable stage. One of the limitations of the method is the production of these intermediates. To achieve the desired pure product, each initial product must undergo changes to be allowed to enter the next stage and eventually become an active peptide. As a result, the overall process of synthesizing an active peptide in this way is often lengthy and difficult [34]. However, using this method, therapeutic peptides with a length of 3 to 20 amino acids have been synthesized and made their way to global markets [35].

The main problems of this method are the insolubility of long peptide chains in organic solvents, long synthesis time, and the amount of chemical waste. Ten years after the invention of the soluble-phase synthesis, the solid-phase synthesis method was invented [36]. This method is based on the reaction of amino acids that, in the presence of insoluble substances, become covered and unreacted in groups. This strategy is actually used to direct the reaction to the desired path. Initially, the first amino acid at the amine end and side-chain groups will cover and then attach to the resin bed via the carboxyl end. After binding, the protecting group washes from the amine terminus and prepares to react with the second amino acid. A coupling compound will be used to bind amino acids to each other. The reactions will then be repeated to obtain the desired product/peptide [37,38].

The simplicity of this method has made it possible to mass-produce bioactive peptides, as it is simpler and faster than the solution-phase synthesis. However, the most important limitation of this method is the massive need for materials to start the process [39]. Therapeutic peptides, such as ziconotide, exanatide, pramlintide, and degarelix, have been synthesized by this method and entered the pharmaceutical market [40]. Around 1992, radio waves were used to further accelerate this method [41]. Today, this method is widely used to produce well-known drugs, such as the antibiotic gramicidin A or the glycoprotein CSF114, which are used for infectious diseases and the clinical diagnosis of multiple sclerosis, respectively [42].

In recent years, the chemical production of peptides by solid-phase method with the help of fluorenylmethoxycarbonyl (Fmoc) as chemical group coating has become one of the most widely used methods. In this method, the protective compound, Fmoc, is used to protect the amino acid side groups. With this action, some groups do not enter the chemical reaction, and the reaction is directed in the desired direction [43]. By and large, the chemical methods mentioned earlier use substances that are environmentally and ecologically hazardous. For example, dimethylformamide and dichloromethane, which are used in chemical methods of synthesizing peptides to protect the amino terminals of amino acids, are very harmful to the environment. The removal of these substances from nature has posed a significant challenge [38].

### 2.3. Recombinant Productions

In the recombinant production of bioactive peptides, the peptide genes are expressed in a specific expression system. Depending on whether the expression system is in vivo or in vitro, recombinant production is divided into two different groups. In the recombinant production of peptides by the in vivo expression system, the desired peptide gene is usually associated with a protein gene, a carrier protein that can be easily purified. One of the advantages of this method is achieving mass production of the desired peptide. Peptides of various drugs have been obtained by this method, including ecallantide and desirudin, which have a length of 60 to 65 amino acids and are expressed in yeast [44,45].

On the other hand, the most advanced recombinant expression system is the expression of bioactive peptides in the in vitro expression system, also called the cell-free system. In the system, all the necessary components for transcription and translation of a peptide gene are present in vitro, and in such an environment, peptide synthesis takes place in the absence of the cell. One of the advantages of this method is the high speed of achieving the desired product [46]. However, due to the cost of this method, it is used for specific peptides and more in the laboratory and research scales. Nevertheless, many pharmaceutical peptides are currently produced from a combination of chemical and recombinant synthesis of pharmaceutical peptides.

In this method, first, a peptide is produced from the desired gene by the biological method. The drug peptide is then chemically modified by chemical methods [47]. One of the peptides that has entered the global medicine market in this way is a peptide called liraglutide, which is used to regulate blood sugar in patients with type II diabetes. This peptide was approved in 2010 by the Food and Drug Administration. It is similar to the hormone glucagon and is recombinantly expressed in yeast, and is chemically added to its amino acid No. 27, which is lysine, a 16-carbon lipid with an average of the amino acid glutamate. This action increases the functional similarity of this peptide to the hormone glucagon [48].

The engineering of bioactive peptides is another topic that has recently attracted a lot of attention. The goal of engineering peptides is to increase their efficacy and stability. Insulin can be cited as an example of the first engineered therapeutic peptide. This hormone is often engineered by substituting one to three amino acids. The purpose of this action is to produce insulin that has a longer effect and can better play the role of natural insulin in the body [49]. Today, a variety of drugs are produced from insulin, each of which has its unique properties as an insulin drug and even its method of administration.

## 3. The Sources of Bioactive Peptides

Potentially bioactive peptides can be extracted or produced from any organism. However, there are a few things to keep in mind when choosing a host. On the one hand, it should be noted that the selection of the target host will ultimately determine the method of extraction and purification of the peptide in question. On the other hand, it should be considered that the production or source of the desired peptide in the host must be so high so that its production/purification will be both economical and problematically worthwhile to go on [11]. In the following section, the different types of industrialized hosts will be described.

### 3.1. Animal Sources

#### 3.1.1. Marine Sources

As mentioned earlier, bioactive peptides can be obtained from a variety of sources. One of these important sources is the use of proteins and body wastes originating from animals to produce bioactive peptides. These peptides are the product of the enzymatic hydrolysis of animal proteins. Blood is an important and rich source of animal protein that is readily available and abundant in slaughterhouses. Other animal sources of bioactive peptides include red meat and aquatic animals. There is growing scientific evidence that many hydrolyzed peptides and proteins derived from marine sources, including mollusks, crustaceans, and fishery wastes (head, intestines, skin, and fins), are capable of promoting human health and preventing chronic diseases [50].

So far, many studies have examined the therapeutic properties of aquatic bioactive peptides (in vitro), and fewer studies have been performed on animal models as well as humans. Huge volumes of fish waste are extracted annually in aquaculture processing companies, which account for up to 75% of the total catch weight. Converting fishery waste into valuable compounds is a suitable solution to reduce environmental pollution and is an optimal use of aquatic waste [51], as the seas cover 70% of the earth’s surface, and their biodiversity is considerably greater than the land surface and accounts for approximately 75% of all living organisms.

Recently, marine peptides have provided a new impetus for the development of pharmacy [52,53]. Peptides discovered from marine organisms stimulate cell death by various mechanisms, including apoptosis, effect on tubulin–microtubule balance, angiogenesis inhibition, antiproliferative effects, and cytotoxic effects. These facts have introduced marine bioactive peptides as a new choice for obtaining new compounds in biomedical research [54]. Because the marine environment has more biodiversity than the terrestrial environment, and due to the unique adaptation of these organisms in dark, cold, and high-pressure environments during the evolution of these organisms, they have been able to express various proteins to overcome these environmental incompatibilities, which can be a huge and unknown source for bioactive peptides. Many bioactive peptides with anticancer potential have been extracted from various marine organisms, such as tonics, sponges, and mollusks [54]. Some of these products, such as aplidine, are now commercially available, and others are in various phases of clinical trials.

About 10,000 types of sponges have been found around the world [55]. To date, a wide range of bioactive peptides have been discovered in just 11 species of sponge. Between them, three genera, which include *Discodermia*, *Petrosia*, and *Haliclona*, can make effective anticancer and anti-inflammatory peptides. Although these compounds have a wide range of biological activities, they are difficult to purify in sufficient quantities for pharmaceutical trials [56]. Jaspamide is a cyclic peptide isolated from sponges of the genera *Jaspis* and *Hemiastrella* and encompasses a large 17-carbon ring and three amino acids that can induce apoptosis in human leukemia cells (HL-60) [56,57]. About nine new cyclic peptides, homophyminics B–E and A1–E1, have been isolated from the *Hamophymia* sponge, which have potent cytotoxic activity. This activity has been reported against several human cancer cell lines. Homophymins A1–E1, which have four amino-6 carbamoyl-2, 3-dihydroxy hexanoic acid structures, have greater potency than the corresponding A-E compounds with the same backbone [58], indicating the importance of chemical contents of bioactive peptides. Geodiamolide H extracted from *Geodia corticostylifera* sponge has been shown to have anticancer activity against breast cancer by disrupting the balance of intracellular actin. Table 1 lists some peptides with several potential therapeutic usages extracted from marine organisms.

Discodermin tetradecapeptides are another group of antiseptic peptides extracted from the *Discodermia* sponge. Phakellistatin peptides isolated from *Phakellia carteri* sponges have also been shown to inhibit the growth of leukemia cells. Another related compound is phakellistatin 13, which is derived from the *Phakellia fusca*. According to recorded observations, this peptide has cell-killing properties against hepatic BEL-7404 cancer cells [74,75]. On the whole, much attention has been paid to the discovery of structural, compositional, and sequence-related properties of bioactive peptides from marine sources.

#### 3.1.2. Milk Products

Dairy products such as milk and cheese are ideal options for extracting animal bioactive peptides. As can be seen from the fundamental role of milk from an early age, where it acts as a source of protein and nitrogen for young mammals, it can be concluded that milk is a valuable substance in terms of protein contents. The proteins in milk have important properties, such as antibacterial, antioxidant, and immunoprotective activities. The number of these properties is increasing every day, and recently, special attention has been paid to the chaperone role of casein proteins in milk. Regarding milk peptides, opioid peptides in milk have been reported to have morphine-like properties on the central nervous system.

Thanks to modern peptide separation and identification systems, tandem mass spectrometry (MS/MS) and high-performance liquid chromatography–mass spectrometry (HPLC–MS), scientists today can obtain peptides with opioid properties from human milk. Other observed activities include ACE inhibitory, mineral-binding, anticarcinogenic, antithrombotic, and cytotoxicity [76]. In addition, lactoferrin (Lf)-protein-derived peptides found in the milk of all mammals play antimicrobial and immunosuppressive roles.

The fermentation of milk protein has also made it possible to access other valuable peptides. Peptides derived from the fermentation of milk by *L. helveticus* LBK-16H, such as the peptides Ile-Pro-Pro, Val-Pro-Pro, Tyr-Pro, and Lys-Val-LeuProi-Val-Pro-Gln, have an ACE inhibitory effect in hypertensive animal models [77]. There are several antioxidant peptides, such as GQGAKDMWR and EWFTFLKEAGQGAKDMWR, derived from donkey milk [78]. Variation in the properties of milk-derived peptides has been shown to depend on factors such as the type of source protein, the hydrolysis method, and even the type of animal host. Today, animal hosts such as camel, mare, goat, sheep, and buffalo are used to extract proteins and peptides from their milk [79]. Some prominent examples of peptides derived from dairy products are listed in Table 2. For a more comprehensive review on BPs from dairy products, see [80].

Other sources for the production of bioactive peptides include hydrolyzed egg white proteins [88], milk, and whey proteins. Whey is one of the byproducts of dairy factories with large quantities and low cost, as well as nutritional properties. Additionally, it has been observed that the antioxidant activity of whey hydrolysates is due to the amino acid cysteine, which participates in the synthesis of glutathione. The composition of whey albumin also chelates heavy metals and reduces the risk of oxidation [89].

#### 3.1.3. Egg Products

From both nutritional and medical perspectives, researchers’ view of egg proteins and peptides extracted from this source is very promising. Today, opinions on eggs are that they are not just a basic nutritional source. For example, a decade ago, the Arg-Val-Pro-Ser-Leu peptide from eggs was chemically synthesized and observed to have ACE inhibitory activity. In addition, the resistance of this peptide in the gastrointestinal tract has raised hopes for the use of these peptides orally [90]. Due to its rich source of amino acids and long shelf life, egg white protein powder (EWPP) is currently used in many sectors of the food and pharmaceutical industries [91]. Major efforts have been made to extract egg peptides through enzymatic digestion. In this way, enzymes such as pepsin, thermolysin, chymotrypsin, alcalase, and trypsin have been used [92].

Approximately 5.3% of the total weight of egg white proteins is the lysozyme [93]. Egg white lysozyme is a rich source of biologically active peptides, such as antimicrobial [94], anticancer, immune regulator, and antihypertensive. Hydrolyzed lysozyme is used in the industry as a natural preservative to prevent the growth of bacteria in meat products, such as sausages, beef, and pork. Heat-denatured lysozyme has been shown to have good antimicrobial activity [95].

#### 3.1.4. Meat Products

Due to the indiscriminate consumption of red meat in some communities, most people today are trying to talk about the disadvantages of this useful food, unaware that this substance is a rich source of essential amino acids, folic acid, vitamin B12, and iron for the human body. Peptides derived from red meat have been shown to exhibit properties such as antioxidant, antimicrobial, and antihypertensive activities [11,96,97]. The ACE-inhibitory effect is one of the classic applications of BPs derived from meat. For the first time, Arihara et al. noticed this activity in peptides derived from porcine skeletal muscle proteins [98]. In addition to porcine, this biological activity has also been observed in beef-protein-derived peptides [99]. Additionally, the antioxidant activity of peptides derived from red meat should not be overlooked. It is found that there is 2700 mg of carnosine per kilogram of pork meat. Carnosine shows well-documented antioxidant activity, which stems from the ability of the carnosine peptide to trap transition metals, such as copper, cobalt, and zinc [100].

Subsequently, after enzymatic hydrolysis (actinase E and papain) of pig protein, Saiga et al. obtained a source of antioxidant peptides [101]. Papain enzyme was also used by Di Bernardini et al. to hydrolyze sarcoplasmic proteins from sarcoplasmic proteins. After fractionation, 10 and 3 kDa fractions showed antioxidant activities [102].

The striated muscles make up about 40% to 50% of livestock meat’s weight. These muscles are made up of fibers of muscle cells. About 55% of red meat proteins contain myofibrillar proteins. These proteins are insoluble in water but are soluble in dilute saline solutions. In the food industry, these proteins have beneficial properties. They comprise relatively large amounts of essential amino acids, which is why they have been considered to be 70% of the nutritional value of meat. These proteins also affect the capacity of the meat emulsion so that 90% of this capacity is due to the presence of these proteins. On the other hand, about 97% of the storage capacity of water is due to the presence of these proteins in the structure of red meat [103]. Sarcoplasmic proteins are given a lot of attention in the production of BPs and makeup about 22% to 25% of the total weight of muscle tissue proteins [104]. Other proteins that receive less attention (due to their lower solubility in water) include connective tissue proteins, such as collagen and elastin. In the food industry, the role of connective tissue proteins is not favorable as they reduce the quality of meat.

#### 3.1.5. Venom Peptidomics: A Cure for the Deathtrap

Scientists’ views on many things have changed today. One of these cases is venom, a composite that mainly includes peptides and other substances [105]. With new uses being discovered for peptides today, some researchers are trying to figure out more applications to access this hidden treasury by identifying the different components of venom [106]. Early efforts in this area focused on the separation of the various parts of venoms and the determination of their active component(s). After separating the active component and performing some other studies, such as sequencing and structuring the active part, it will be possible to study more applications of the discovered component. Efforts in this area have intensified to the point that researchers are trying to build a library of ‘expressed sequence tags’ related to the venom glands of various animals. Although these studies are expensive, they are worth the benefits that will be discovered in the future [107]. The studies in this field are numerous and varied, and Table 3 presents a brief classification overview of different families of venom peptides along with their applications.

#### 3.1.6. Other Animals

In addition to the aforementioned sources, some other animals can be a significant source of BPs. For example, bacterial antimicrobial peptides are called bacteriocins, which contain neutral or positively charged peptides and are secreted by a variety of Gram-negative and positive bacteria. These peptides are not a factor in defending against viral infection, but help bacteria kill other bacteria in the competition for environmental supplements [113].

Most bacteriocins target the cell membrane with hydrophilicity or hydrophobicity, while some also inhibit the biosynthesis of biopolymers or the activity of enzymes. The peptides are not synthesized by ribosomes and instead undergo complex step-by-step compression reactions derived from peptide synthetases. Large nonribosomal (NRPS) peptides are often composed of nonprotein amino acids, including D-type amino acids, hydroxy acids, or other unusual compounds, and exhibit a wide range of inhibitory and functional mechanisms [114]. Bacteriocins are resistant to heat, low pH, weak organic solvents, cold, ice, and salts and are therefore applicable to food protection systems. Isolation and purification of these compounds are necessary to determine the exact mechanism of their inhibitory activity on food spoilage bacteria and foodborne bacteria. Bacteriocins are generally sensitive to human intestinal proteases, making them a valuable resource for food preservation without potential harmful effects on human health [115]. Additionally, antimicrobial peptides can be extracted from the hemolymph of insects or outside their bodies. Insects in the face of microorganisms secrete different antimicrobial peptides because they can detect different types of invasive organisms and secrete the appropriate antimicrobial peptide. Antimicrobial peptides in primitive organisms are an alternative to primary responses [116].

More attention is also being paid to BPs derived from marine microorganisms. Some marine bioactive compounds are produced by microbes that coexist with marine species. Marine actinomycetes are a source of secondary bioactive compounds that have anticancer and antimicrobial activity. Cyclomarins A, B, and C are three ring-shaped heptapeptides isolated from marine actinomycetes, such as *Streptomyces* sp. Cyclomarin A is composed of three common amino acids and four unusual amino acids and has shown anti-inflammatory and anticellular activity in laboratory studies [117,118]. Salinamides A–E are types of peptides that have been extracted from *Streptomyces* sp. Salinamides A and B are two bioactive compounds with large rings that have local anti-inflammatory and antimicrobial activity against Gram-negative bacteria and are used in the treatment of tissue inflammation and some infections. Salinamides C, D, and E are small BPs with anti-inflammatory activity. The structure of type D is similar to that of A, but contains valine residues at the isoleucine site of type A. Salinamides E and C are also shown as a single-ring peptide [119].

Additionally, amphibians have a high level of defense system that includes innate and acquired immunity. In this group of organisms, the skin is protected by innate immunity mediated by macrophages, neutrophils, complement-mediated lysis, natural killer cells, and secreted antimicrobial peptides. Peptides of this group are synthesized in the granular glands of the skin. Due to environmental stimuli or damage to the sympathetic nerves, the peptides are activated and the contents of the glands are secreted to the surface of the skin [120]. Antimicrobial peptides in this group mostly have an alpha-helix structure. It has been shown that *Rana* frogs secrete the ranalexin antimicrobial peptide, which has a cyclic structure with disulfide bridges [121].

### 3.2. Plant Sources

Traditionally, more attention has been paid to peptides derived from animals than plants. Nevertheless, it should be noted that plant proteins are rich sources of proteins without saturated fatty acids that can carry useful ingredients. Recently, certain activities have been discovered in plant-derived peptides that can perform important functions in humans. Antidiabetic, immunomodulatory, antimicrobial, hypocholesterolemic, opioid, antihypertensive, and antioxidant activities are of these benefits.

BPs are derived from plant sources such as plant proteins and/or directly extracted from them. Plant sources of BPs, due to their cost-effectiveness and lower immunogenic effects, have recently received more attention from experts in this field. Most plant proteins are incomplete due to a lack of essential amino acids, but wheat germ protein contains essential amino acids and is therefore categorized as valuable as animal proteins, such as meat or chicken eggs [122]. From an immunological point of view, the skin and its components are the first level of defense against invading microorganisms. The same rule is more or less true for plants. Antimicrobial peptides in plants also help plants in the early stages of the fight against invading microorganisms. Antimicrobial peptides with beta plates have been identified in plants, with two groups of these well-studied peptides including thionin and defensins. Thionins are the first identified peptides from plants that play a significant role in protecting plants against invading bacteria. This group of peptides is toxic to a variety of Gram-negative and Gram-positive bacteria, and even mammalian bacteria [123]. Although plant-derived peptides work to inhibit the growth of various types of microorganisms, there are more detailed classifications, such as antivirals, antifungals, and antiparasitics. It is believed that the antibacterial activity of peptides is due to the presence of motifs rich in positive amino acids and the amphiphilic nature of the peptide sequence [124,125]. Ultimately, these properties of antimicrobial peptides help them penetrate the bacterial membrane to create pores and eliminate the disturbing bacteria by altering homeostasis. To help readers’ knowledge of this article, the table below (Table 4) lists the types of antimicrobial peptides found in plants. Additionally, in the following sections, several images are used to explain the mechanism of antimicrobial activity. Although many plant antifungals or antivirals have more than 50 amino acid sequences, such as 2S albumin-like from *Malva parviflora* [126,127], lipid transfer proteins (LTPs) [126,128], and puroindolines [129,130], they are omitted here because they do not belong to the peptide category.

## 4. Medicinal Applications and Proposed Mechanism of Actions of BPs

### 4.1. Antioxidant Activity of BP and Its Mechanism of Action

In recent years, the trend of technological developments in human societies has caused fundamental changes in human lifestyle. By reducing the physical activity of people in the community, which can be considered a special type of stress, the incidence of some diseases, including cardiovascular complications and different types of cancers, has increased in people in developed and developing societies. Looking through the literal definition of stress, it is the general and nonspecific response of the organism to maintain homeostasis against any factor that threatens or impairs the body’s compensatory abilities [140]. In its general definition, stress is a factor that interferes with a person’s physical and mental balance, causes psychosomatic problems, and reduces a person’s efficiency in various aspects of life [141].

Oxidative stress is a change in the balance between pro-oxidants and antioxidants. The production of some free radicals such as superoxide can be physiologically beneficial, but oxidative stress occurs when the balance between the production of reactive oxygen/nitrogen species (ROS/NOS) and the antioxidant defense system is upset. Oxidative stress, therefore, changes the balance between pro-oxidants/antioxidants in favor of pro-oxidants, potentially leading to biological damages. Diseases associated with ROS production include cancer, Parkinson’s, and Alzheimer’s [142] diseases. Oxidative stress can cause serious damage to important cellular macromolecules, including lipids, nucleic acids, and proteins. In biological systems, the production of free radicals of ROS is inevitable, and the body partially neutralizes their harmful effects by designing antioxidant defense mechanisms. The most important components of the enzymatic antioxidant defense system include the enzymes superoxide dismutase, glutathione peroxidase, and catalase. Antioxidant enzymes, which are responsible for detoxifying free radicals or repairing antioxidant molecules, are an indicator of stress levels in cells or tissues. In addition to the primary defense barrier created by antioxidant enzymes, the second defense barrier is created by small molecules (antioxidants) that react with free radicals to produce less dangerous radical compounds [143].

BPs have a strong antioxidant activity against free radicals and other reactive species. These antioxidant peptides contain 5–16 amino acids [144]. The mechanism by which peptides exert their antioxidant effects has not been fully elucidated, although various studies have shown that hydrolyzed peptides and proteins prevent enzymatic and nonenzymatic oxidation by removing free radicals and chelating metal ions. Several peptides have been found in protein constituents that have antioxidant capacity, and their biological activities have been extensively studied. Although the energy of free radicals (such as hydroxyl) is high, in general, all 20 amino acids found in proteins can have internal interactions with free radicals. Food-derived antioxidant peptides are safe and healthy compounds with low molecular weight, low cost, high activity, and easy absorption. The antioxidant properties of peptides are mostly related to their composition, structure, and hydrophobicity [144,145].

#### 4.1.1. Effect of Amino Acid Contents on Antioxidant Activity of Peptides

The presence of some amino acids and their position in the peptide sequence has an important effect on their antioxidant activity [146]. Aromatic amino acids, such as tyrosine, histidine, tryptophan, and phenylalanine, and hydrophobic amino acids, such as valine, leucine, methionine, glycine, and alanine, are essential for the antioxidant role of the peptide. The higher oxidation of peptides compared with free amino acids is attributed to their unique chemical and physical properties by the amino acid sequence itself. In a study, the His-Gly-Pro-Lue-Gly-Pro-Lue antioxidant peptide, the presence of two replicate sequences, Gly-Pro, and the placement of Lue in the carboxylic position and His at the amine end increased the free radical scavenging property [147]. The presence of hydrophilic amino acids such as proline, alanine, valine, and leucine in the N position and the amino acids tyrosine, valine, methionine, leucine, isoleucine, glutamine, and tryptophan in the C-terminal position was associated with the antioxidant properties of peptides [148]. Additionally, fat-soluble free radicals (peroxyl radicals) produced during the oxidation process of unsaturated fatty acids are neutralized by hydrophobic amino acids such as leucine, valine, alanine, and proline [149]. Amino acids such as histidine, tyrosine, methionine, and cysteine inactivate free radicals by giving them protons. Aromatic amino acids (phenylalanine, tryptophan, and tyrosine) convert free radicals into stable molecules by giving them electrons [150].

#### 4.1.2. Effect of Peptide Size on Antioxidant Activity

In addition to a peptide sequence, the molecular weight of peptides can affect their antioxidant activity [147,151]. Research has shown that the antioxidant activity of corn gluten hydrolyzed protein is related to its concentration and molecular weight. The antioxidant activity of peptides with a molecular weight of between 500–1500 Daltons is stronger than that of peptides with a molecular weight of higher than 1500 Daltons or lower than 500 Daltons [152]. In some cases, the higher antioxidant power of smaller peptides compared with large chain peptides was attributed to their easier access to free radicals and a more effective removal of these radicals [153]. However, it has been repeatedly shown that the higher the degree of hydrolysis, the lower the antioxidant activity of the peptides. This is due to the further breakdown of peptides into free amino acids that have little or no antioxidant activity [146].

#### 4.1.3. The Role of Hydrophobicity of Peptides in Their Antioxidant Activity

Most food-derived antioxidant peptides include hydrophobic amino acids such as valine or leucine at the N-terminal and proline, histidine, tyrosine, tryptophan, methionine, and cysteine in their sequence. Hydrophobic amino acids such as valine or leucine can increase the affinity of peptides in the fat phase, thus facilitating access to free radicals produced in the fat phase [153,154].

### 4.2. Mechanism of Antimicrobial Activity

**Figure 3:**
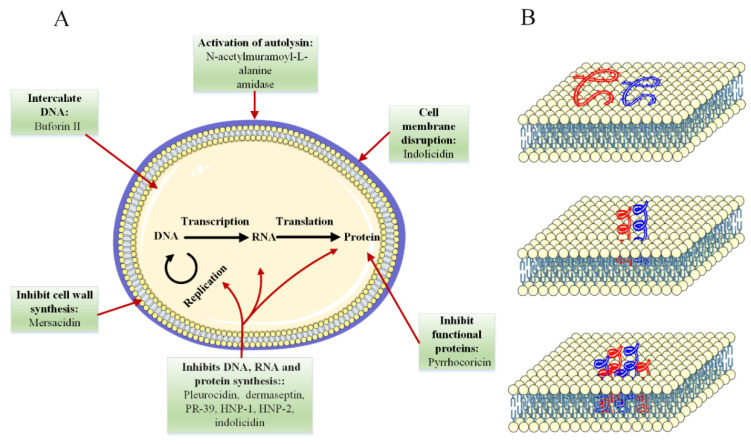
(**A**) Types of mechanisms of action of antimicrobial peptides on bacterial cells. (**B**) How antimicrobial peptides penetrate the cell membrane.

In the last two decades, many peptides with antibacterial, antiviral, and antifungal activities have been identified in both vertebrates and invertebrates, which form an important part of the host’s innate immune system. In most cases, the mechanism of action of antimicrobial peptides appears to be different from that of conventional antibiotics (see Figure 3). For this reason, these peptides are very interesting as new drugs to fight infectious agents [155]. Thus, antimicrobial peptides have opened a new chapter in the sciences, which has attracted the attention of many scientists and researchers, especially since they are much simpler in structure than proteins, which simplifies the study of the function–structure relationship and makes it possible to construct them by nonbiological ways (such as chemical synthesis).

The factors of the effectiveness of these biologically active peptides as antimicrobial agents depend on structural properties (e.g., peptide size, amino acid composition, or charge) [156]. However, antimicrobial peptides have some common features. Most antimicrobial peptides are composed of fewer than 50 amino acids, 50% of which are hydrophobic [157]. Among these peptides, those with essential amino acids (lysine and arginine) have the highest antimicrobial properties [158,159]. Additionally, cationic and amphipathicity amino acids are important structural features for the antimicrobial activity of these BPs [160,161]. These peptides are also referred to as cell-penetrating peptides, protein transport domains, membrane sequences, or trojan peptides. Today, these peptides are used to transport many membrane materials. Recent studies have shown that these peptides can transport a wide range of drugs, proteins, liposomes, and nanoparticles into animal cells [162]. In 1988, a membrane-permeable peptide was discovered from the Tat protein of the HIV-I virus with tree sequences. This peptide was found to be able to cross the membrane of cultured cells and accumulate in the nucleus [163,164].

It has been concluded that in the case of some antimicrobial peptides, although the peptides reduce the growth of harmful microbes, they do not directly interact with the target microbes or microorganisms, but do so with the help of the host immune system [165]. For example, milk protein hydrolyzate has been shown to stimulate the host immune system. These activities include stimulating the proliferation of the natural killer cell, stimulating macrophage phagocytosis, and encouraging the expression of many antibodies, cytokines, and chemokines [166].

The anti-inflammatory function of peptides is usually related to their antimicrobial activity [167]. Inflammation is the response of the immune system to harmful stimuli (which can be invasive agents or damaged tissues) that are necessary to protect living organisms. In other words, inflammation is a complex biological response of host cells, vascular tissue, proteins, and other mediators to eliminate the primary causes of cell damage, tissue hemorrhage, and necrotic cells, which ultimately leads to the elimination of infection and treatment. During an inflammatory response, by increasing blood flow and vascular permeability, immune system components can escape from the blood vessels to the affected area, resulting in five symptoms that may indicate inflammation: redness, heat, swelling, pain, and loss of function.

Inflammation is normally controlled and limited on its own. Inflammatory mediators are activated only in response to harmful stimuli and have a short lifespan, and when the harmful agents are removed, they are destroyed or inactivated. Additionally, at this time, the acute inflammatory response is over, infection is removed, and damaged tissue is repaired. In addition, various anti-inflammatory mechanisms are activated. If the causative agent cannot be eliminated quickly, it may lead to chronic inflammation that can have serious pathological consequences. At the end of the inflammation, several different regulatory mechanisms are activated: (1) inflammatory mediators that are short-lived are destroyed or inactivated; (2) leukocyte migration stops; (3) the permeability of the vessels decreases and returns to normal; (4) the expression of proinflammatory molecules decreases, and conversely, the expression of anti-inflammatory molecules increases, which causes the transfer of monocytes instead of neutrophils. Monocytes clean dead and damaged tissues, and tissue repair begins [168,169].

The production of natural antimicrobial agents by phagocytes has long been known. These antimicrobial peptides provide the first line of defense against pathogens in eukaryotic organisms and are generally effective against bacteria, fungi, and viruses. In addition to the direct killing of microbes, these compounds also participate in processes related to inflammation and innate and acquired immunity. Antimicrobial peptides, which are innate immune mediators, increase phagocytosis and trigger the release of prostaglandins. They also neutralize the shock effects of liposaccharides caused by bacteria. These peptides transport and accumulate immune cells at the site of inflammation, induce angiogenesis, and heal wounds. The production of cytokines is also affected by these peptides [113]. Antimicrobial peptides also have a chemotactic role [170]. All of these actions eliminate the cells of bacteria. The results showed that these compounds are bactericidal at high concentrations of mg/mL and have a safety regulatory role at lower concentrations [171].

Given all of the above, antimicrobial peptides are probably involved in all stages of host defense. In addition to enhancing the immune response, these compounds prevent uncontrolled inflammation by suppressing proinflammatory responses. Despite the specific overlap, the antimicrobial peptides interact with each other, complementing each other to guide effective cells to the site of inflammation and modulate the local immune response [172]. Phagocytes, neutrophils, and monocytes are absorbed via alpha-defensins, human neutrophil peptides 1HNP1-3, and beta-defensins such as human β-defensins 2hBD3 and 3hBD4, while mast cells are adsorbed via HNP1-3, LL-37, and 4B. In addition, hBD1 and hBD3 are chemotactic for immature dendritic cells and memory T cells.

The combination of these peptides and cytokines at the site of injury will help these immature dendritic cells to mature and enable them to process antigens and migrate to nearby lymph nodes, where antigens are present. Antimicrobial peptides indirectly play a chemotactic role by inducing or increasing chemokine secretion. For example, LL-37 induces the release of interleukin-8 by lung epithelial cells, and human defensin HNP1-3 induces the activation and degranulation of mast cells. In addition, these human peptides increase neutrophil invasion, stimulate further transcription, and produce interleukin-8 by bronchial epithelial cells [173].

Antimicrobial peptides have a dual property: on the one hand, they protect the host against harmful pathogens through antimicrobial activity, and on the other hand, they protect the host from the harmful effects of excessive inflammatory responses. In other words, these peptides stimulate the production of proinflammatory cytokines, increase the accumulation of dendritic cells and monocytes at the site of injury, and increase phagocytosis and maturation of dendritic cells, while simultaneously protecting the organism from the harmful effects of an inflammatory response. As a result, these peptides have both proinflammatory and anti-inflammatory roles.

Cathelicidin is an important family of cationic peptides. In humans, the cathelicidin gene encodes an inactive precursor protein that finally matured to active 37-amino acid peptide (IL-37). It causes a balance between proinflammatory and anti-inflammatory signals. Such peptides can inhibit the host’s harmful proinflammatory responses without losing the beneficial innate defense [174]. Another example, α-melanocyte stimulating hormone (α-MSH), is a neuropeptide that belongs to the melanocortin family with anti-inflammatory effects and shares several properties with antimicrobial peptides. Recent studies indicate the direct antimicrobial activity of this peptide against fungi and pathogenic bacteria. It reduces the concentration of proinflammatory mediators and thus protects the brain and peripheral organs from inflammatory disorders. Therefore, α-MSH is an anti-inflammatory peptide with antimicrobial properties [175].

### 4.3. Antihypertensive Peptide and Its Mechanism of Action

In 2000, there were 972 million cases of hypertension in the world, and this number is expected to reach 1.56 billion by 2025 [176]. The angiotensin-converting enzyme plays an important role in regulating and increasing blood pressure. This enzyme catalyzes the transformation of inactive angiotensin I (decapeptide) to activate angiotensin II (octapeptide), which is a strong vasoconstrictor. Angiotensin II also has a regulatory effect on the enzyme cellular lipoxygenase, which accelerates the oxidation of low-density lipoprotein (LDL) and is associated with atherogenesis. It is also an inhibitor of bradykinin, a potent vasodilator.

Clinical studies have shown that ACE inhibitors significantly reduce mortality in patients with myocardial infarction or heart failure [177]. Captopril and enalapril inhibitors are used for hypertension, but they have many different side effects, including coughing, changes in taste, pimples, and edema, so there is a great deal of interest in using natural antihypertensive peptides [178]. In addition to milk proteins [179], other sources of antihypertensive peptides have been investigated. These sources include egg protein, mainly ovalbumin [180]; meat protein [98]; beef hemoglobin [181]; gelatin [182]; fish skin protein [183]; and several plant proteins, such as soy [184], sesame [185], broccoli [186], buckwheat [187], and transgenic rice protein [188]. In most cases, for the body to use the antihypertensive peptides, these molecules must be absorbed intact through the intestines and enter the bloodstream.

### 4.4. Mechanisms of Opioid Activity

Although pain is an important sign that there is a problem somewhere in the body, it is an unpleasant feeling that is often accompanied by severe and destructive stimuli. Chronic pain is associated with high levels of depression and anxiety. Additionally, in some physical conditions, reduced physical activity due to the generation of pain causes other diseases, such as obesity and heart harm. Chronic pain originates in the brain and/or spinal cord and is often difficult to manage [189]. Opioid drugs are currently used to relieve such pain, despite being associated with undeniable side effects. Pain is also one of the most important challenges in the management and/or treatment of cancer, and it has been seen that the psychological effects of pain in cancer patients have a negative effect on their recovery [190,191]. The probability of pain in advanced stages of cancer is close to 70% to 80% [192]. Pain is also seen in 90% of patients who have experienced cancer metastasis [193].

For these reasons, understanding the principles of pain and its management is critical for this group of patients. Pain is divided into two categories in terms of location: peripheral pain and nerve pain. Peripheral pain is pain that originates outside the central nervous system, including superficial pain, deep pain, or visceral pain. Nerve pain may be due to a pathophysiological condition of the central nervous system, such as deep disturbance or secretion of microbial–chemical substances and irritation due to heat or cold. Central pain, neuritis, neuralgia, and causalgia are types of nerve pain [194].

So far, various therapeutic measures have been used to control pain. Common therapeutic measures include the administration of non-narcotic and narcotic analgesics (opioids). The types of drugs used are [195]:

(a) Muscle relaxants such as probantin and belladonna group such as atropine;
(b) Vascular dilators such as papaverine hydrochloride or nitroglycerin;
(c) Anti-inflammatory drugs such as indomethacin, ibuprofen, and phenylbutazone;
(d) Non-narcotic analgesics such as aspirin and acetaminophen;
(e) Narcotic analgesics such as Demerol and methadone hydrochloride.

Most drugs reduce blood pressure and respiratory depression, bradycardia, and confusion [196,197]. Scientific methods of acute postoperative pain relief introduce the use of both narcotic and non-narcotic drugs along with the use of nerve blocks as the method of choice for complete postoperative pain relief. Oral and injectable methods (intramuscular, intravenous, and subcutaneous) for dermal or mucosal absorption or central or peripheral nerve blocks or without catheter placement are a variety of ways to administer the drug [198].

As mentioned, one of the ways to control pain is to use narcotics. These drugs are generally peptides between 5 and 80 amino acids, which generally have two sources, endogenous and exogenous [199]. Drug peptides bind to their receptors on the surface of nerve cells, triggering a signal that ultimately reduces pain. According to studies, most drug peptides act as agonists. In addition to pain, these peptides have been shown to reduce stress levels. The internal sources of opioid peptides are usually in the form of either hormones (secreted by the glands) or a neurotransmitter that is secreted by nerve cells and acts on the terminals of other cells [200].

Enkephalin was the first known endogenous peptide. Many endogenous peptides have been shown to have a conserved Tyr-Gly-Gly-Phe sequence at the end of their N-terminal [201,202]. Exorphins or exogenous opioid peptides with morphine-like activity enter the body from food sources, or in emergencies through drugs and supplements. Among the available food sources, dairy products are the best source for exorphins due to the similarity of the sequence of peptides derived from them to endogenous opioid peptides. Some observations suggest that the product of enzymatic digestion of dairy proteins, especially milk, can bind to opioid receptors on the cell surface. For example, the Arg-Tyr-Leu-Gly-Tyr-Leu-Glu peptide derived from bovine milk casein alpha protein has been shown to have narcotic activity [203]. It is important to note that peptides resulting from the digestion of digestive enzymes can be easily administered orally to humans [204]. Interestingly, opioid peptides of animal origin generally bind to µ receptors, and peptides of plant origin bind to ẟ receptors [205].

### 4.5. Mineral-Binding Peptides

Proteins interact with ions through their amino acid side chain. For example, alpha-casein and beta-casein interact with divalent and trivalent cations, such as calcium. In addition to proteins, peptides also have the ability to bind minerals. For example, casein-derived phosphopeptides, also known as casein phosphopeptides, have this activity [206]. These phosphopeptides are involved in maintaining calcium, phosphorus, and other mineral elements in solution in intestinal pH. This activity is due to the presence of the amino acid phosphorylated serine, which can make salts with minerals, such as calcium. Enzymatic digestion of milk produces a diverse group of these peptides [207]. The type of amino acid composition present in the phosphorylated region plays an essential role in the amount of calcium-binding activity in this group of peptides [208]. These peptides are also effective in preventing tooth decay, osteoporosis, insomnia, and hypertension. Animal studies have shown a positive effect of these peptides on calcium absorption. A group of researchers has shown that fermentation of whey protein with *Lactobacillus holoticus* is effective in the proliferation of osteoblasts in vitro. There have also been other reports of the increased bioavailability of iron in rat models [209].

### 4.6. Blood-Lipid-Lowering Effect

Hyperlipidemia, especially high cholesterol, is one of the most important risk factors for cardiovascular disease. Many studies show that soy-derived peptides can lower blood cholesterol levels in animal models of hepatotoxicity as well as in humans. Soy-rich diets have become one of the most effective dietary treatments for high cholesterol, although the mechanism has not yet been fully elucidated. It is believed that soy peptides derived from protease actively cut cholesterol traveling in the gut and thus reduce cholesterol uptake [210]. Other research has shown that hydrophobic peptides derived from soy proteins are able to interact with bile acids, thereby increasing the excretion of fatty acids in the feces [97,209].

It has been indicated that LPYPR and IAVPGEVA peptides derived from soy glycinin protein, which have structural similarity with endostatin and VPDPR, showed a cholesterol-lowering effect. These peptides inhibit 3-hydroxy-3-methylglutaryl-coenzyme, a reductase, which is a key enzyme during the biosynthesis of cholesterol [97]. Milk is another important source of BPs with a cholesterol-lowering effect. In 1999, Nagaoko et al. discovered a cholesterol-lowering peptide from digested beta-lactoglobulin hydrolase. Hydrolyzed plant protein with cholesterol-lowering activity such as soy and hydrolyzed *Brassica carinata* proteins have also been reported [211]. Such effects from aquatic animals, such as sardine [212] and zebrafish [213], have been shown on blood lipids. It has been shown that peptides that are lower in proportion to the amino acids methionine, glycine, lysine, and arginine are better able to cause hyperlipidemia. However, bovine casein protein, which has a higher proportion of these amino acids, especially methionine and glycine, raises cholesterol levels [214].

### 4.7. Antiobesity Effect

Peptides can affect the absorption of nutrients in the intestines, especially the small intestine, thereby reducing appetite. Many studies have shown that peptides derived from dietary proteins can send satiety signals to the brain and thus prevent the consumption of more foods [214]. Casein-derived peptides have been shown to regulate eating in the body by activating the cholecystokinin A (CCK-A) receptor [215].

### 4.8. Antidiabetic Activity

A wide range of plant-derived peptides can help diabetics through a variety of pathways. The pathways that have been studied so far include inhibitory properties on alpha-amylase, dipeptidyl peptidase IV, glucose transporter system, and mimicking insulin activity [216].

### 4.9. Antiaging Peptides

During the aging process, the production of extracellular matrix proteins, such as collagen, fibronectin, elastin, and laminin, decreases, and their breakdown increases. In addition to protecting the cell structure, the extracellular matrix is effective on cellular behaviors, such as proliferation and differentiation. Such functions are controlled by small peptides derived from the breakdown of extracellular matrix proteins called matrikine [217,218]. Following the destruction of the extracellular matrix, the elasticity of the skin gradually decreases, and the first lines of aging and wrinkles appear [219,220].

The aging process of the skin is controlled by internal and external factors. External factors include exposure to ultraviolet radiation, environmental air pollution, and smoking. Exposure to ultraviolet light increases reactive oxygen species (ROS), disrupts collagen synthesis, and induces collagenase production and enzymes that break down proteins in the extracellular matrix, thereby causing cell DNA damage, and ultimately destructs the skin integrity. Interior factors include the formation of large amounts of reactive oxygen species during cellular metabolism and genetic factors that cause the destruction of extracellular matrix proteins and reduced blood flow, and the function of skin cells is reduced [221].

Gradually, with the discovery of more functions of BPs, it was seen that these molecules are able to rejuvenate the skin and increase its repair power. Today, peptides are used to stimulate collagen, heal wounds, and smooth out skin wrinkles and for antioxidant, antibacterial, and skin whitening effects [222,223,224]. Cosmetic peptides can affect skin freshness through several mechanisms. These pathways can be used to signal peptides that modulate collagen levels, such as Lys-Thr-Thr-Lys-Ser [225]; reduce IL-6 secretion and stimulate collagen and elastin synthesis, such as palmitoyl tetrapeptide-7 [226]; stimulate human skin fibroblasts and increase skin angiogenesis, such as Val-Gly-Val-Ala-Pro-Gly peptide; and inhibit tyrosine enzyme activity, such as Nonapeptide-1 [227].

Some peptides act as inhibitors of neurotransmitters. The mechanism of action of neurotransmitter-inhibitory peptides similar to botulinum neurotoxin type A under the brand names Botox^®^ [228] and/or Dysport^®^ [229] is the most widely used skin beauty product, which weakens muscle contractions and thus reduces wrinkles by inhibiting the signal path of neurotransmitters in nerve–muscle connections. In the use of these peptides, the most important challenge being studied today is their transfer from the skin layers to the lower and living parts of the skin.

## 5. Cyclic Peptides: One Step Ahead of Linear Peptides

Some studies have suggested that linear peptides have not been able to meet expectations in some applications. For example, the most important drawback of linear peptides is their low stability, which can cast a shadow on their therapeutic applications. In recent decades, a new gate of research has been opened to design cyclic peptides to overcome some of the challenges of linear peptides. Cyclic peptides have been shown to be less susceptible to proteolysis [230] and have a higher receptor binding capacity than their linear counterparts [231]. It is believed that the cyclization of peptides ultimately reduces their spatial vibrations, provides a large surface area for interacting with the target, and consequently strengthens their binding to the target molecule and also increases metabolic stability [232]. Although in nature, similar to the examples given in the section on marine peptides, there are many sources of cyclic peptides, solid-phase synthesis of them has now become a common method in many laboratories around the world [233]. Nearly three decades ago, the production cost of cyclic peptides was very high, almost prohibitive for mass production, but much effort in the chemical synthesis of peptides, development of new methods for the purification of the peptides, and significant reduction in the price of amino acids compared with the past have led to more attention to cyclic peptides [233]. The table below shows a number of cyclic peptides with their function and source (Table 5).

## 6. Use of Computer-Based Techniques in Peptide Research

Computer-based methods, such as proteomic and peptidomic studies, are very helpful in researching peptides. Using computer-based techniques, it is possible to predict the production of peptides from specific dietary proteins. With this method, the selection of enzymes, proteins, and hydrolysis products, as well as the study of the secondary structure and physical and chemical properties of the produced peptides, would be possible. The classic method for identifying and processing BPs involves in vitro digestion and chromatographic purification of the hydrolysis product. After the bioactivity test, the peptide sequence will be usually identified. Most of the time, this process continues with the confirmation of the biological activity of the chemically synthesized peptide sequence.

The main problem in the classical method is the low yield and limitation in the number of peptide samples that are studied at one time. On the other hand, proteomics-based methods are based on high-efficiency protein digestion and techniques for predicting peptide activity using computer-based systems (in silico) that provide biological and chemo-metric information about the desired peptide sequence. The key steps in this method are as follows: first, the protein databases are examined to select the desired proteins with known amino acid sequences. The proteins are then digested in silico using the appropriate proteolytic enzymes for the selected protein. Peptides produced in silico are then examined for structural properties and potential biological activities, including toxicity and allergenicity [245]. A convenient and useful list of the different databases for structural and physical properties of peptides was recently created [246].

## 7. Conclusions and Future Perspectives

BPs can be identified as specific amino acid sequences that have beneficial physiological effects. Some of these peptides are inactively buried in the structure of proteins and are activated by extraction from parental proteins. Technology for the production of BPs including protein hydrolysis by microbial enzymes, plant or animal enzymes, and fermentation using different amino acids to produce peptides with separate or multiple biological functions provided a promising way to reach a better quality of life. Today, BPs are known as products of protein hydrolysis of various foods. These peptides play a variety of biological roles, one of the most important of which is antioxidant activity. The inverse relationship between antioxidant activity and the occurrence of diseases has been proven in several studies. The results showed that the antioxidant power of hydrolyzed protein was lower than standard antioxidant solutions, such as ascorbic acid and ethylenediaminetetraacetic (EDTA), but due to the fact that natural antioxidants are usually used as alternatives in larger quantities due to their lower potency than synthetic antioxidants, in this case too, higher doses can be recommended for greater effectiveness.

Marine BPs also showed a variety of physiological functions, such as immune stimulation, hypotension, antidiabetic, antioxidant stress, antiobesity, skin protection, and wound healing. These effects have been investigated in animal and human models, and the results indicate the promising effect of peptides as beneficial compounds in the production of food–drug compounds and other drug supplements. Regarding BPs originating from the oceans, it is worth mentioning a few suggestions: (I) Investigation of different extracts of different marine species such as sponges and algae will identify compounds with medicinal properties in order to produce new drugs. (II) Bioactive compounds with marine sources are a good alternative to land-based drugs that have shown drug resistance in humans. (III) Marine environments account for the largest area of the planet relative to land and contain unknown and undiscovered compositions that confirm the need for further research.

Past and current research on antimicrobial peptides has shown that these compounds have great potential for use in the food and medical industries. Continuous discoveries of new antimicrobial peptides and understanding of the process, biological systems involved in the synthesis, safety, and regulation of antimicrobial peptides have paved the way for advances in this field with an emphasis on practical applications in the industry. Genetic engineering or chemical modification of bacteriocins to improve their functional properties has been considered in recent years, which has led to significant development in bacteriocin technology. While most classical research has focused on finding antimicrobial peptides on prokaryotic sources, there is ample evidence that most life forms produce small peptides with antimicrobial properties. These include not only bacterial but also hydrophilic proteinlike compounds in eukaryotes, such as mammalian defensin and cathelicidins, frog magainin, insect thanatin, and plant thionin.

In addition, a large number of antimicrobial peptides are known that have not yet been properly used, and their applications have not satisfactorily been discovered. Therefore, more work needs to be done to investigate the applications of these compounds, especially in the new sectors of the food and medical industries. For example, there is increasing knowledge about the role of antimicrobial peptides in reducing the prevalence of some cancers, especially colon cancer. The exact mechanism of action is not known, but it is likely due to the control of mutagenic compounds in the intestine by direct binding to carcinogens or inhibition of the microbes that produce these agents. Therefore, there is a high potential for the use of antimicrobial peptides, and more research in this field can lead to promising results that have significant effects in the food and medical industries.

## Figures and Tables

**Table 1:** Marine bioactive peptides.

Peptide	Organism	Function	Ref.
Peptide extracts	*Bacillariophyceae*	Antihypertensive/antioxidant	[59]
Peptide extracts	*Discodermiu kiiensis*	Antimicrobial	[60]
Azonazine	*Aspergillus insulicola*	Anti-inflammatory	[61]
Wewakazole	*L. majuscula*	Anticancer	[62]
Mirabamide A-C-D	*Sponges*	anti-HIV	[63]
Aplidine	*Aplidine*	Anticancer	[64]
Arenastatin A	*Dysidea arenaria*	Anticancer	[65]
Aurilide	*Dolabella auricularia*	Anticancer	[66]
Didemnin	*Trididemnum* sp.	Anticancer	[67]
Dolastatin	*Dolabella auricularia*	Anticancer	[68]
Geodiamolide H	*Geodia* sp.	Anticancer	[69]
Homophymines	*Homophymia* sp.	Anticancer	[58]
Jaspamide	*Jaspis* sp., *Hemiastrella* sp.	Anticancer	[70]
Kahalalide F	*Elysia rufescens, Spisula polynyma*	Anticancer	[65]
Keenamide A	*Pleurobranchus forskalii*	Anticancer	[56]
Mollamide	*Didemnum molle*	Anticancer	[71]
Phakellistatins	*Phakellia carteri*	Anticancer	[72]
Tamandarins A and B	*Didemnum* sp.	Anticancer	[73]

**Table 2:** Some recent examples of milk peptides with their applications.

Precursor Protein	Peptide Sequence	Bioactivity	Ref.
Hydrolysates of camel milk protein	KDLWDDFKGL and MPSKPPLL	Antidiabetic	[81]
Hydrolysates of camel milk protein	LPVPG	Antidiabetic	[82]
Hydrolysates of camel milk protein	FLQY, FQLGASPY, ILDKEGIDY, ILELA, LLQLEAIR, LPVP, LQALHQGQIV, MPVQA, and SPVVPF	Antidiabetic	[83]
Hydrolysates of camel milk protein	KFQWGY, SQDWSFY, and YWYPPQ	Inhibition of cholesterol esterase	[84]
Bactrian camel milk hydrolysate	RLDG QGRPRVWLGR, TPDNIDIW LGGIAEPQVKR, and VAYSDDGENWTEYRDQGAVEGK	Antioxidant	[85]
Fermented camel milk (*Leuconostoc lactis*)	MVPYPQR	ACE inhibitor	[28]
Fermented goat milk (*Lactobacillus plantarum 69*)	ND	ACE inhibitor	[86]
Hydrolyzed goat milk	ND	Antimicrobial activity	[87]

**Table 3:** Different families of venom peptides.

Classification	Example	Host	Applications	Ref.
Bradykinin potentiating peptides	TsTX-Ka and TsTX-KO	*Bothrops jararaca*	Hypotensive effects, ACE inhibitor	[108]
	BPPs	*Tityus serrulatus* *Bothrops jararaca*	ACE inhibitor	[109]
Antimicrobial peptides	IsCTs	*Opisthacanthus madagascariensis*	Antimicrobial Cytolytic activity	[110]
Hormonelike peptides	Mini-Ins	*Conus geographus*	Insulin-like activity	[111]
Therapeutic peptides	Ziconotide	*Conus magus*	Pain killer	[112]

**Table 4:** Plant-derived antimicrobial peptides.

Plant	Peptide	Peptide Size	Biological Activity	Ref.
*Hevea brasiliensis*	Heveins	43 residues, 4.7 kDa	Antibacterial and antifungal	[127]
*Phaseolus vulgaris*	ND	2.2 and 6 kDa	Antibacterial and antifungal	[131,132]
*Brassica napus*	Peptides	ND	Antiviral	[133]
*Capsella bursa-pastoris*	Shepherins	28 residues	Antibacterial and antifungal	[134]
*Higher plants*	Thionins	45–47 residues	Antibacterial	[126,127,128]
*Oldenlandia affinis*	Cyclotides	28–37 residues	Antibacterial, Antifungal, Insecticide, Nematicide	[126,135]
*Phytolacca americana*	PAFP-S	36–37 residues	Antibacterial	[136]
*Triticum aestivum*	Alpha-1-purothionin	45 residues	Antibacterial	[137]
*Triticum aestivum*	Defensins	5 kDa	Antibacterial and antifungal	[138]
*Benincasa hispida*	Hispidulin	5.7 kDa	Antibacterial and antifungal	[139]

ND: not determined.

**Table 5:** Different well-known cyclic peptides along with their application and source.

Name	Source	Application	Ref.
Gramicidin S	*Bacillus brevis*	Antibiotic activity towards Gram-negative and Gram-positive and even several pathogenic fungi.	[234,235]
Tyrocidine	*Bacillus brevis*	By antibiotic action, it can disrupt the cell membrane function.	[236]
Plitidepsin	*Aplidium albicans*	Antitumor, antiviral, and immunosuppressive activities.	[237]
Cyclosporin A	*Tolypocladium inflatum*	As a calcineurin inhibitor, it can decrease the function of lymphocytes.	[238]
Alisporivir	Chemically synthesized from ciclosporin	Inhibits cyclophilin A, and it is believed that it may have a potential effect on Alzheimer’s disease and hepatitis C.	[239,240]
Romidepsin	*Chromobacterium violaceum*	By apoptotic activity, it has an anticancer activity on many types of malignant cell lines.	[241,242]
Ziconotide	*Conus magus*	Acts as an analgesic agent; strong pain killer.	[243]
Vancomycin	*Amycolatopsis orientalis*	A board range antibacterial compound that is used in many bacterial infections.	[244]

## Data Availability

Not applicable.
